# The exposure to and health effects of antimony

**DOI:** 10.4103/0019-5278.50716

**Published:** 2009-04

**Authors:** Ross G. Cooper, Adrian P. Harrison

**Affiliations:** Division of Physiology, Birmingham City University, 704 Baker Building, Franchise Street, Perry Barr, Birmingham B42 2SU, UK; 1 Section for Biochemistry and Physiology, Department of Animal and Veterinary Basic Sciences, Faculty of Life Sciences, Copenhagen University, 1870 Frederiksberg C, Denmark

**Keywords:** Antimony, environment, exposure, health, occupation

## Abstract

**Context:**

This minireview describes the health effects of antimony exposure in the workplace and the environment.

**Aim:**

To collate information on the consequences of occupational and environmental exposure to antimony on physiological function and well-being.

**Methods:**

The criteria used in the current minireview for selecting articles were adopted from proposed criteria in The International Classification of Functioning, Disability and Health. Articles were classified from an acute and chronic exposure and toxicity thrust.

**Results:**

The proportion of utilised and non-utilised articles was tabulated. Antimony toxicity is dependent on the exposure dose, duration, route (breathing, eating, drinking, or skin contact), other chemical exposures, age, sex, nutritional status, family traits, life style, and state of health. Chronic exposure to antimony in the air at levels of 9 mg/m^3^ may exacerbate irritation of the eyes, skin, and lungs. Long-term inhalation of antimony can potentiate pneumoconiosis, altered electrocardiograms, stomach pain, diarrhea, vomiting, and stomach ulcers, results which were confirmed in laboratory animals. Although there were investigations of the effect of antimony in sudden infant death syndrome, current findings suggest no link. Antimony trioxide exposure is predominant in smelters. Mining and exposure via glass working, soldering, and brazing are also important.

**Conclusion:**

Antimony has some useful but undoubtedly harmful effects on health and well-being and measures need to be taken to prevent hazardous exposure of the like. Its biological monitoring in the workplace is essential.

## INTRODUCTION

Antimony (Sb) (atomic number 51; atomic mass 121.75 g/mol; density 6.684 g/cm^3^; melting point 631°C) occurs naturally as a sulphide ore, stibnite (Sb_2_S_3_) and valentinite (Sb_2_0_3_). The traditional method of treating the ore is to roast it with charcoal or coke and collect the volatile oxide fume (Sb_4_O_6_) from which pure antimony is refined.[1] A noxious gas stibine (SbH_3_) is formed when antimony reacts with nascent hydrogen.[2] The trichloride (SbCl_3_) is very toxic as it dissociates into its oxide and chlorine.[1] The element is used in alloys, as a constituent of paint pigments, and in rubber compounding. Very pure antimony is used to make certain types of semiconductor devices such as diodes and infrared detectors. Workers exposed to airborne dust can have respiratory problems. Exposure can also occur via contaminated water, food, and soil contact. In 1916, antimony exposure was associated with lead intoxication, with symptoms including headache, abdominal pain, constipation, colic, distaste for food, loss of appetite, small mouth ulcers with salivation, dizziness, loss of weight, albuminuria, and glycosuria.[3] The lack of importance of antimony compounds in terms of their mutagenic, carcinogenic, and teratogenic risks in pregnant women has negated the need for studies of the like.[4] Exposure to antimony concentrations of 9 mg/m^3^ of air will result in eye, skin, and lung irritation.[5] Long-term exposure to antimony in smelting plants may result in the formation of antimoniosis, a particular form of pneumoconiosis.[6] Chronic exposure will potentiate lung, heart, and gastrointestinal diseases. The effects on health are inducement of vomiting and eye and mucous membrane irritation. Stibine is a hemolytic agent. In some cases, cardiac arrhythmias and mild jaundice may occur necessitating treatment with intramuscular dimercaprol.[2] McCallum[1] describes the effect of antimony in potentiating antimony spots, heart disease, pneumoconiosis, and lung cancer. Other authors suggest an increased mortality from lung cancer and associated non-malignant respiratory heart diseases in workers exposed to antimony.[7] Antimony in conjunction with other metals may biologically alter several cellular defence mechanisms thus potentiating carcinogenesis.[8] Reproductive disorders and chromosome damage may be associated with chronic antimony exposure with consequent mutagenic and oncogenic potential and should be avoided in pregnancy and in patients with hepatic, renal, or cardiovascular disease.[9] Measurement of antimony and particulate matter is via samples collected onto a cellulose acetate filter (pore size, 0.8 *µ*m) at an air flow rate of ca. 21/min. Absorption spectrophotometry is used to analyse the concentration of antimony. The workplace exposure limit is 8-h Time Weighted Average (TWA) of 0.5 mg/m^3^.[2]

In 1467, at Beverley, Yorkshire, 19^th^ and 20^th^ century laws dealing with air pollution were anticipated.[10] Penalties were imposed on illegal kiln constructions in an attempt to alleviate the air pollution[10] because antimony ores are frequently associated with arsenic or lead and exposure of workmen working with imported stibnite ore has been reported.[1] Antimonate ore has been reported to induce toxic effects and activate some protective mechanisms, including phagocytosis, immune responses, and inhibition of proteases.[11] Historically, antimony became a fashionable cure among the wealthy and was approved as a medicine in 1666.[12] Paracelsus described antimony-disease as a specific condition caused by external factors for which specific remedies were needed.[12] Drugs made from antimony were administered as purges.[13] The successful curative effects of antimony on the sick King Louis XIV of France in a concoction called *vin émétique* disposed of any controversy concerning its efficacy.[13] Although in 1566 and 1615 Paris banned the use of antimony as it was regarded as poisonous, it was officially reinstated by the parliament in 1666.[1]

The ancient medical uses of antimony spanned nearly 600 years since its introduction as an internal treatment in the 14^th^ century.[1] Antimony administered as a tartar emetic in the form of one and two grains in a child and adult, respectively, may lead to death.[14] It should therefore be taken with copious amounts of water and fomentations placed over the chest two to three times per day, followed by a cool, moist pack.[14] This was explained as reducing the exhausting effects of the drug. Goose livers were conditioned with deliberate antimony administration in Strasbourg, Germany, an area which specialised in goose-liver pies (*paté de foie gras*). The handling of the birds was horrific, often with their feet secured in planks and their eyes gorged out. Antimony was then force-fed. This was to satisfy the satietary whims of French, English, and American gourmands.[14] The treatment for antimony poisoning consisted of one teaspoon of ground mustard/powdered alum in warm water (emetic) repeatedly in order to induce vomiting until the stomach was void of its contents.[14] If necessary, manual stimulation of the back of the throat with a finger or feather ensured vomiting. Milk or albumen could be administered if the poison had irritating effects. This was followed by strong tea, a decoction of oak bark, or an infusion of tannin.[14]

The aim of this minireview was to subjectively assess the literature on the material concerning the health benefits of antimony and to classify it according to the source and publication date.

## MATERIALS AND METHODS

The criteria used in the current minireview for selecting articles to be included were both theoretically and practically motivated and adopted from proposed criteria in The International Classification of Functioning, Disability and Health – ICF.[15] These criteria were as follows:

1. Articles were chosen only with internationally recognised impact factors greater than 0.10.
2. Articles were chosen based on the impact of lifestyle, stress, and/or environmental factor/s predisposing antimony exposure.
3. Criteria for selection of the literature used included yes–no responses to the appropriateness of methodology; adequacy of subject numbers; specificity of sex and/or age of subjects, and statistically significant response rates to survey questionnaires.
4. The time frame used was principally 1900–2008 inclusive, although articles of extreme importance from earlier decades were used where appropriate.
5. A multifactorial overview of the factors eschewed concerning zinc exposure was elucidated. It was presumed that collective articles detailing known factors of usage were not necessarily correlated with functionality and health.
6. Compilation of materials for the minireview started with the published literature or easily accessible academic research.
7. The articles were accessible from on-line sources, including PubMed and Medline.

## RESULTS

The proportion of utilised and non-utilised articles by date is shown in Table 1. There seemed to be a dearth of investigations spanning the period 1900–1980, although some articles were published in sources unobtainable through requests at the British library and some had no impact factor and were excluded.

**Table 1:** Selection results for articles on the influence of antimony (Sb) and its health effects on the body

Time period	Total # journal articles	Total # health brochures and website	Inclusion # journals	Exclusion # journals
1900–1960	2	0	2	0
1961–1970	3	0	0	3
1971–1980	14	0	0	14
1981–1990	26	0	5	21
1991–2000	60	3	25	35
2001–2010	59	1	29	30
Total	164	4	61	103
		(4 utilised)	(57 utilised)	(103 non-utilised)

### Public health effects of antimony

The Agency for Toxic Substances and Disease Registry[16] has composed a comprehensive account of the public health effects of antimony, including health effects by route of exposure (inhalation, oral, and dermal exposure), toxicokinetics (absorption, distribution, metabolism, and excretion), biomarkers of exposure, and effect, among other chapters. It is not the scope of the current review to repeat facts contained therein. Clearly, antimony toxicity is dependent on the exposure dose, duration, route (breathing, eating, drinking, or skin contact), other chemical exposures, age, sex, nutritional status, family traits, life style, and state of health. One may also be exposed to antimony alloys used in lead storage batteries, solder, sheet and pipe metal, bearings, castings, type metal, ammunition, and pewter.[16] Lead and antimony concentrations peak at 4060 and 119 *µ*g/m^3^, respectively, in the air following shooting in an indoor range.[17] Antimony has also been used widely as a flame retardant in fabrics and brake linings.[1] Antimony may enter the environment as a consequence of mining and processing of its ores and in the production of antimony metal, alloys, antimony oxide, and combinations with other substances. Antimony released from smelters may remain in particulate quantities in the air, some of which reaches the soil during rainfall where it attaches strongly to particles containing iron, manganese, or aluminium.[16] Generally, the concentration of antimony in the soil is very low < 1 part per million parts of soil (ppm). Extremely high soil concentrations have been found at hazardous waste sites at 109–2550 ppm. From there, it easily enters plants and grazing animals. On average, ca. 5 *µ*g antimony is consumed with food everyday, with 0.2–1.1 ppb being found in meats, vegetables, and seafood. Airborne concentrations of antimony average < 1–170 ng/m^3^ air depending on proximities to smelters or mines, which make it >1,000 ng/m^3^ air. The Occupational Safety and Health Administration in the USA and UK have set a limit of 0.5 mg/m^3^ of antimony in workroom air, assuming that workers complete an 8-h shift and 40-h working week.[16] In post-mortem studies on deceased smelter workers, antimony may not act systemically along with other metals in a multiple fashion due to non-significant differences in tissue concentrations in the liver and kidneys by comparison with controls.[18]

The concentration of riverine antimony is <5 parts in 1 billion parts water (ppb) and it does not accumulate in fish and aquatic organisms. There has, however, been a report expressing concern of the accumulation of antimony in reef fish and suggestions were made for The Agency for Toxic Substances and Disease Registry to analyse future samples with detection limits lower than health screening values, speciation of seaweeds being performed, and an investigation of the potential effects of catching and eating sea food being carried out.[19] The US Environmental Protection Agency (USEPA) has set a limit of ca. 145 ppb antimony in lakes and streams. In drinking water, this level should not exceed 0.006 ppm and environmental spills or discharges of ≥2267.96 kg must be reported.[20] However, antimony in mining effluent may increase to 8 ppb, usually combined with soil particles. Thankfully, antimony does not leach from drinking water pipes.[16]

Chronic exposure to antimony in the air at levels of 9 mg/m^3^ may exacerbate irritation of the eyes, skin, and lungs.[16] Long-term inhalation of antimony can potentiate pneumoconiosis, altered electrocardiograms, stomach pain, diarrhea, vomiting, and stomach ulcers, results which were confirmed in laboratory animals.[16] Long-term exposure in experimental animals has shown an increase in the hepatic malfunction and blood changes.[20]

The limitation existing in the study of antimony includes the lack of studies of the influence of the metal on lung cancer in human subjects. Urine, fecal, and blood tests designed to measure antimony levels do not indicate how much antimony one has been exposed to or whether one will experience adverse health effects. Testing may require extremely sophisticated equipment, much of which is not available in GP surgeries.[20]

### Case reports of antimony exposure

Quantification of urinary antimony levels was performed by both quadrupole (Q) and sector field (SF) inductively coupled plasma mass spectrometry (ICP-MS). The limits of detection were (Q and SF) 1.09 and 0.43 ng/L for antimony. The urinary median values observed in healthy subjects from central Italy were 60.8 ng/L for antimony.[21] Metals measured in casual (spot) urine specimens by ICP-MS demonstrated that peripheral arterial disease (PAD) risk increased sharply at low levels of antimony and remained elevated beyond 0.1 *µ*g/L. Although other heavy metals like cadmium are more important, antimony may be associated with PAD.[22] Antimony toxicity was described in a 19-year-old man with a history of alcohol abuse who ingested a 10 ml bottle of “Soluto Vital” (tartar emetic, 50 mg/ml), produced in Guatemala for treatment of alcohol abuse. He presented 60 min after ingestion with severe vomiting, abdominal cramps, diarrhea, weakness, and orthostasis. Initial laboratory evaluations were remarkable for creatinine of 2.5 mg/dl, potassium 6.1 mEq/L, and 60% haematocrit. He was given activated charcoal, IV saline, and antiemetics. Over the next 48 h, his creatinine normalized to 1.1 mg/dl and the hematocrit returned to 53%. His urine had an antimony concentration of 1200 mcg/L (normal = < 10 mcg/L).[23]

### Antimony, sudden infant death syndrome (SIDS) and infancy

The role of antimony compounds in SIDS was determined via hepatic biopsies, although there was no difference between the two categories of cause of death, SIDS or those who had died of an identified disease.[24]

The toxic gas hypothesis proposes exposure to stibine (antimony trihydride) generated from microbial contamination of cot mattress materials as a possible cause of SIDS as a consequence of cholinesterase inhibition.[25] However, the study showed that stibine gas or soluble antimony compounds are not capable of inhibiting cholinesterase activity at toxicologically relevant concentrations.[25]

Normal cot environment conditions are non-optimal for volatilization of antimony by *Scopulariopsis brevicaulis* and that Sb_2_O_3_ in cot mattress polyvinyl chloride (PVC) is not bioavailable.[26] *In vitro* experiments showed that there was release of stibine and phosphine, hydrides of antimony oxide fire retardant, and phosphorus plasticisers from PVC mattress covers, which had been treated with these substances.[1] Body fluids (urine, saliva) and proprietary domestic detergents/sterilizing fluids markedly enhanced leaching of antimony from PVC.[27] Release of antimony was also enhanced at both low and high pH and by elevated temperature. The extent of antimony leaching did not correlate well with the PVC content of this element. Ingestion of antimony released from PVC could account for the high variability associated with the reported detectable levels of antimony in liver from both SIDS and other infants.[27,28]

The ability of fungi to produce volatile arsenic and antimony compounds in pure culture was examined using *S. brevicaulis*, reported as an inhabitant of PVC cot mattress covers.[29] Although antimony levels above the baseline sensitivity of the analytical technique were detected in four (out of 24) of the samples analysed, the concentrations recorded were too low to be reliably interpreted as evidence for volatilization.[29]

Urinary antimony was assayed using ICP-MS in 201 urine specimens collected at different ages throughout the first 2 years of life from 122 term and 26 pre-term infants.[30] Absolute antimony concentrations varied widely between infants, being below the laboratory detection limit of 0.02 *µ*g/L in 7% of the samples, below 0.5 *µ*g/L in 90.5%, and above the reference value of 1 *µ*g/L reported for non-occupationally exposed UK populations in 4%.[30]

The prevalence of SIDS has fallen since 1988 probably due to the change in preferred posture of infants in cots from prone to supine positions. Indeed, the detection of antimony in the hair of healthy babies suggests that there is no link between its toxic accumulation and SIDS.[1]

### Antimony trioxide

Antimony trioxide treatment is associated with increased apoptosis associated with induced reactive oxygen species (ROS) as well as differentiation markers. When the buffering capacity of the cell is decreased by depleting glutathione, ROS production and apoptosis is enhanced.[31] Antimony trioxide is primarily used as a flame retardant in rubber, plastics, pigments, adhesives, textiles, and paper. Antimony potassium tartrate has been used worldwide as an anti-shistosomal drug. Pentavalent antimony compounds have been used for the treatment of leishmaniasis. Both trivalent and pentavalent antimony compounds are generally negative in non-mammalian genotoxicity tests, while mammalian test systems usually give positive results for Sb(III) and negative results for Sb(V) compounds.[32]

A study of 23 male workers assigned to different fire retardant treatment tasks in the car upholstery industry vs. a control group of 23 healthy non-exposed males revealed that the group of inspection operators who directly handled a mixture containing Sb_2_O_3_, with a significantly higher proportion of workers in this group, had oxidative DNA damage compared with controls.[33]

International occupational standards limits for antimony exposure are 500 *µ*g/m^3^. The range of 42 personal exposures was 0.01–0.55 *µ*g Sb/m^3^, while 24 area samplings ranged from < 0.01 to 1.45 *µ*g Sb/m^3^.[34] The mean urinary Sb levels at the beginning (*n* = 39) and at the end of the shift (*n* = 39) were 0.31 ± 0.25 *µ*g/L and 0.35 ± 0.29 *µ*g/L, respectively, without any significant statistical difference. No correlation was found between personal Sb_2_O_3_ exposure and the difference in urinary antimony levels at the beginning and at the end of the work shift on the day the flame retardant was utilised.[34] This lack of correlation could be due to low airborne Sb_2_O_3_ levels and antimony dietary intake, estimated as 3 *µ*g/day in UK.[34]

The concentrations of the immunoglobulin (Ig) G subclasses, IgE, interleukin-2, interferon-gamma, and interleukin-4 in sera obtained from workers exposed to antimony through antimony trioxide manufacture were determined and compared with those of control subjects.[35] The serum levels of IgG1, an Ig involved in host defence against many microbial infections, were significantly lower in the sera of Sb-exposed individuals than in the controls. The serum concentrations of IgE, an Ig mediating allergic hypersensitivity, were also lower in the Sb-exposed workers than in the controls. The levels of interleukin-2 and interferon-gamma, multifunctional cytokines for T-cell-mediated immunity, were lowered in the factory workers.[35] A significant positive correlation between IgG4 and urine Sb levels was found among the Sb-exposed workers, indicating a possible role of IgG4 in Sb-mediated pulmonary or skin pathogenesis.[35] A study of exposure to antimony trioxide fumes in a brazing rod manufacturer resulted in a systemic eruption of follicular papules and pustules.[36]

### Antimony and glass workers

Heavy metals have been shown to alter the mechanism and release of lysosomal enzymes. The activities of lysosomal glycohydrolases were determined in order to evaluate the asymptomatic toxic effects of low levels of exposure to arsenic and antimony in art glass workers by a fluorimetric assay. Secretion of lysosomal glycohydrolases was increased by antimony (225%) at a concentration of elements (200 *µ*g/L).[37]

The use of antimony as a fining agent in art glass manufacture results in exposure thereof in workers, showing that antimony was higher in batch mixers and low in makers-formers.[38] One study determined that there was no definite correlation between antimony exposure and the risk of lung cancer, but rather to nickel and copper.[39]

### Antimony and worker health

There is an association of lung cancer with antimony exposure, although smoking greatly exacerbates the chances thereof.[40] The toxicology of antimony and its compounds is known from three sources: its medicinal use over centuries, studies of process workers in more recent times, and, currently, studies of its presence in modern city environments and in domestic environments. Gross exposure to antimony compounds over long periods, usually the sulphide (SbS_3_) or the oxide (Sb_2_O_3_), has occurred in antimony miners and in antimony process workers. The working conditions in antimony processing have improved markedly over the last 30 years and the workforce has been much reduced in numbers following automation of the process.[1] The health of antimony workers was a concern of Sir Thomas Oliver (1853–1942), who was distinguished in the field of occupational medicine. Antimony appears to have fascinated him, but he underestimated its toxic effects on the process workers in concluding that they were healthy and that there were no industrial hygiene problems in the process.[1]

Searches of the literature revealed a variety of sources of antimony associated with city life. Copper refinery inhalable aerosol fractional analysis showed no detectable levels of antimony.[41] As a potential role of antimony as a confounder in human health studies related to arsenic in drinking water, tube-well water concentrations of antimony and arsenic were analysed in the Pabna region of Bangladesh by ICP-MS using USEPA method 200.8. All 245 water samples had antimony concentrations <1 *µ*g/L.[42] Silver, copper, nickel, and antimony were significantly higher in living areas of jewellers' homes compared with control homes (*P* ≤ 0.04). Ventilation measures did not effectively reduce metal concentrations in jewellers' homes.[43] Non-exhaust emissions from road vehicles are out of control, including particles from brake wear, tyre wear, road surface abrasion, and resuspension. Brake dust particles may contain antimony.[44] Antimony exposures on blood and urine levels in the optoelectronic workers were determined by ICP-MS. Blood indium and urine gallium and arsenic levels in the 103 workers were significantly higher than that in the 67 controls during the follow-up period. In regression models, the significant risk factors of exposure were job title, preventive equipment, Quetelet's index, sex, and education level.[45]

In an investigation of the trace element content in myocardial and muscular biopsy samples with clinical, haemodynamic, and histological diagnosis of idiopathic dilated cardiomyopathy (IDCM), there was a large increase (>10,000 times for mercury and antimony) in myocardial specimens.[46] Antimony was 12,000 times (19,260 ng/g vs. 1.5 ng/g) greater in IDCM.[46]

A 4 h exposure to >50 *µ*M antimony trichloride (SbCl_3_) could induce micronuclei formation in cultured Chinese hamster ovary (CHO-K1) cells, human bronchial epithelial (BES-6) cells, and human fibroblasts.[47] Although apoptosis and DNA fragmentation was not found in cells immediately following 4-h SbCl_3_ treatment, DNA fragmentation was detected in CHO-K1 cells after 4-h SbCl_3_ treatment and a 16 h or more post-incubation in fresh medium by 1.5% agarose gel electrophoresis.[47]

Antimony is suspected to be carcinogenic to humans although the experimental and epidemiologic data are limited.[48] Arsenic and antimony can be found as environmental cocontaminants, resulting in coexposure to man.[49] Chromosomal mutagenicity induced by arsenic (III) was significantly suppressed by antimony (III), suggesting the necessity to identify putative environmental cocontaminations of antimony in the regions contaminated with arsenic and to determine the impact of antimony coexposure on arsenic genotoxicity and carcinogenicity in man *in vivo*.[49]

Antimony (III) oxide (by inhalation) has been shown to cause lung cancer in female rats.[50] Antimony is less widely present in the environment. There is evidence that in mammals antimony is not detoxified via methylation but it still remains unclear what mechanism is responsible for antimony's genotoxicity.[50]

### Antimony in common food sources

A dietary assessment of exposures of consumers to 30 elements of the UK 1994 Total Diet Study compared with those from previous UK Total Diet Studies and those from other countries showed low exposures to antimony.[51]

Antimony, measured by hydrogen generation/ICP-MS (detection limit = 1 *µ*g/kg) demonstrated a dietary intake of ca. 0.252 *µ*g/kg/week (WHO tolerable daily intake = 6 *µ*g/kg/week). The Principle source of antimony included milk. The study suggested that antimony ingestion in the diet is insignificant.[52]

### Antimony and autoemissions

The antimony emission factors originating from automobiles were ca. 32 *µ*g Sb/braking/car for PM10 (emission factors of brake abrasion dusts of 5.8 mg/braking/car) and 22 *µ*g Sb/braking/car for PM2.5 (emission factors of brake abrasion dusts of 3.9 mg/braking/car).[53]

### Antimony and plastic

Antimony poses both acute and chronic health effects in drinking water. Antimony concentrations in bottled waters ranged from 0.095 to 0.521 ppb, well below the USEPA maximum contaminant level of 6 ppb. Summertime temperatures inside of cars, garages, and enclosed storage areas can promote antimony leaching from polyethylene terephthalate (PET) bottled waters. Microwave digestion revealed that the PET plastic used by one brand contained 213 ± 35 mg Sb/kg plastic.[54] Antimony residues, a result of the use of a polycondensation catalyst in the production of PET oven-proof trays, were analysed in ready-to-eat meals. Microwave and conventional oven cooking caused a distinct increase in the concentration of antimony in food and ready meals of 0–17 and 8–38 *µ*g/kg, respectively.[55]

### Antimony and soil/mines

Antimony in soil sampled at various locations in the vicinity of mines showed a concentration of antimony of 100.6–5045 mg/kg (WHO limit of exposure = 36 mg/kg).[56] In the United Kingdom, five former mining and smelting sites were investigated and found to have levels of total antimony of up to 700 mg/kg, indicating high levels of contamination that could be potentially harmful.[57] However, this level of antimony was found to be biologically unavailable over a wide range of pH values, indicating that antimony is relatively unreactive and immobile in the surface layers of the soil, remaining where it is deposited rather than leaching into the lower horizons and contaminating the ground water.[57]

A biomonitoring study of 218 residents (age 1–89 years) was carried out for a putatively increased absorption of antimony from the environment.[58] The results did not show a correlation between the mercury and antimony contents in the soil of the housing area and those in the urine and hair. This suggested minimal exposure to the heavy metals.[58]

A study showed that no elevated content of antimony could be detected in 24-h urine, blood, or scalp-hair samples from the study participants geogenically exposed to antimony.[59] The results did not show a correlation between the antimony contents in the soil of the housing area and those in the urine, blood, or hair.[59] Blood contents of the control and study groups were 0.57 and 0.48 *µ*g Sb/L, respectively. The detection limit for urine and blood was 0.5 μg Sb/L and that for scalp hair was 0.005 mg Sb/kg. The rate of transfer of antimony from the soil to humans in the exposure case described seemed to be very low.[59]

Part of the northern Palatinate region in Germany is characterised by elevated levels of antimony in the soil due to the presence of ore sources and former mining activities.[60] Antimony has been found in at least 114 different ores.[1] There was no correlation between the antimony contents in the soil of the housing area and those in the urine and hair. Except for antimony in scalp hair, age tended to be associated with internal exposures to antimony.[60] The arsenic and antimony contents in scalp hair were positively correlated with the 24-h arsenic excretion in the urine. Antimony in scalp hair was not correlated with seafood consumption, as was arsenic in scalp hair and in urine.[60]

### Antimony and soldering

Soldering and brazing operations, utilising testing by gravimetry of a filter with collected sample that was mineralized with concentrated and atomic absorption spectrometry, revealed a TWA of fume concentration of antimony as Sb <0.035 mg/m^3^.[61]

## DISCUSSION

Antimony use is widespread in the world and workers need to be completely protected by being provided with overalls and respirators, with regular monitoring of air levels and inspection of air extraction systems. Its biological monitoring in the workplace is essential. One interesting study suggested sweat excretion studies of antimony.[62] Additionally, as a test for screening and controlling the effects of visceral leishmaniasis, chemotherapy by solusurmine in renal failure patients is suggested.[62] One could add that the urinary excretion of antimony is another important monitoring tool as it is related to the intensity of exposure. Indeed, after 8-h exposure to 500 *µ*g Sb/m^3^, the increase of urinary antimony at the end of the shift is equivalent to 35 *µ*g/g creatinine.[63] Others suggest monitoring antimony levels in hair samples using an instrumental neutron activating analysis.[64] Despite the concerns expressed by Sir Thomas Oliver and the health of persons exposed to antimony,[65] antimony currently has some potentially useful medical applications, including its use as a tartar emetic against lung tumor cell lines.[1]
